# Endoplasmic Reticulum Stress Links Oxidative Stress to Impaired Pancreatic Beta-Cell Function Caused by Human Oxidized LDL

**DOI:** 10.1371/journal.pone.0163046

**Published:** 2016-09-16

**Authors:** Valérie Plaisance, Saška Brajkovic, Mathie Tenenbaum, Dimitri Favre, Hélène Ezanno, Amélie Bonnefond, Caroline Bonner, Valéry Gmyr, Julie Kerr-Conte, Benoit R. Gauthier, Christian Widmann, Gérard Waeber, François Pattou, Philippe Froguel, Amar Abderrahmani

**Affiliations:** 1 Univ. Lille, CNRS, Institut Pasteur de Lille, UMR 8199 - EGID, Lille, France; 2 Service of Internal Medicine, Centre Hospitalier Universitaire Vaudois and Lausanne University, Lausanne, Switzerland; 3 Univ. Lille, Inserm, CHU Lille, U1190 - EGID, Lille, France; 4 Department of Stem Cells, Andalusian Center for Molecular Biology and Regenerative Medicine, Seville, Spain; 5 Department of Physiology, Lausanne University, Lausanne, Switzerland; 6 Department of Genomic of Common Disease, Imperial College London, London, United Kingdom; Universidad Miguel Hernandez de Elche, SPAIN

## Abstract

Elevated plasma concentration of the pro-atherogenic oxidized low density lipoprotein cholesterol (LDL) triggers adverse effects in pancreatic beta-cells and is associated with type 2 diabetes. Here, we investigated whether the endoplasmic reticulum (ER) stress is a key player coupling oxidative stress to beta-cell dysfunction and death elicited by human oxidized LDL. We found that human oxidized LDL activates ER stress as evidenced by the activation of the inositol requiring 1α, and the elevated expression of both DDIT3 (also called CHOP) and DNAJC3 (also called P58IPK) ER stress markers in isolated human islets and the mouse insulin secreting MIN6 cells. Silencing of Chop and inhibition of ER stress markers by the chemical chaperone phenyl butyric acid (PBA) prevented cell death caused by oxidized LDL. Finally, we found that oxidative stress accounts for activation of ER stress markers induced by oxidized LDL. Induction of *Chop/CHOP* and *p58IPK/P58IPK* by oxidized LDL was mimicked by hydrogen peroxide and was blocked by co-treatment with the N-acetylcystein antioxidant. As a conclusion, the harmful effects of oxidized LDL in beta-cells requires ER stress activation in a manner that involves oxidative stress. This mechanism may account for impaired beta-cell function in diabetes and can be reversed by antioxidant treatment.

## Introduction

The progressive dysfunction and destruction of pancreatic beta-cells is a key feature of the onset and progression of type 2 diabetes (T2D) [1–4]. The resulting decline in beta cell function is characterized by a loss in cell number caused by an increased apoptosis rate and defective insulin production and secretion from the remaining beta cells [1–4]. It has been suggested that in the context of systemic insulin-resistance, low grade inflammation, chronic excess of cholesterol and of metabolic fuels including the non-esterified fatty acid palmitate and glucose, trigger beta-cell damage over time, especially in genetically predisposed individuals [1–4]. Furthermore, elevated plasma levels of oxidized low density lipoprotein cholesterol (LDL) act as additional potential diabetogenic stressor and increase the risk for associated cardiovascular diseases [5]. Indeed, specific antibodies against oxidized LDL have been reported in patients with T2D [6]. High oxidized LDL levels are commonly found in the obesity-associated metabolic syndrome [7] and further increase throughout the development of T2D [8]. Importantly, several studies have reported the presence of receptors for oxidized LDL in both human and rodent islet beta-cells [9–12]. The deleterious effects of human oxidized LDL on beta-cell function have been evidenced by *in vitro* experiments. The copper-mediated oxidation of LDL provokes similar modification within the particles to those occurring in human [13]. This oxidation is therefore commonly used to mimic the effects of oxidized LDL [11,14–16]. The administration of mildly oxidized LDL (2 mmol/l) to isolated human and rat pancreatic islets, as well as into insulin producing beta-cells decreases both production and secretion of insulin, and ultimately kills beta-cells by inducing apoptosis [11,14–16]. The adverse effects of oxidized LDL rely on mechanisms that involve both oxidative stress and induction of cAMP responsive element modulator (CREM, also called ICER) [16]. However native LDL at similar cholesterol concentration (2 mmol/l) does not trigger harmful effects on beta cells [15,16].

The endoplasmic reticulum (ER) might play a key role in mediating adverse effects of oxidized LDL on beta-cells. First, ER stress is involved in beta-cell dysfunction and death caused by several diabetogenic stressors including chronic hyperglycemia and hyperlipidemia [17–20]. Second, the ER stress is closely linked to oxidative stress [21,22]. We have previously shown that oxidative stress is induced by oxidized LDL, contributing to beta-cell death and impaired insulin expression [16]. In contrast, treatment of beta-cells with the antioxidant N-acetylcystein (NAC) prevented beta-cell decline caused by oxidized LDL. In addition, high density lipoprotein cholesterol (HDL) has antioxidant property and antagonizes the harmful effects of oxidized LDL [11,14–16]. Last, oxidized LDL triggers ER stress signaling transducers that includes the eukaryotic translation initiation factor 2 alpha kinase 3 (EIF2AK3, also called PERK), endoplasmic reticulum to nucleus signaling 1 (ERN1, also called IRE1α), and activating transcription factor 6 (ATF6) in vascular cells [23]. Thus, we postulated that the ER stress may act as a possible mediator for the deleterious effects of oxidized LDL on pancreatic beta-cells.

## Materials and Methods

### Materials

The 4-phenylbutyric acid (PBA) compound, N-acetylcystein (NAC) and thapsigargin were obtained from Sigma-Aldrich (St. Louis, MO). Rabbit monoclonal anti-phospho-PERK (Thr980) and anti-phospho-eIF2A (Ser51) were purchased from Cell Signaling Technology (Danvers, Ma), while the antibodies against beta tubulin, BiP, PERK and phospho-Ire1α were from Santa-Cruz Biotechnology (CA, USA). The small interfering RNAs (siRNAs) against Chop were ON-TARGET*plus* SMARTpool siRNA from Dharmacon.

### Lipoprotein preparation

Blood collection from human healthy donors, plasma LDL (LDL density, 1.063) and HDL fractions were prepared exactly as described [15,16]. Oxidation of LDL particles was done by incubation of 1 mg LDL protein/ml PBS with 5 μmol/l CuSO_4_ at 37°C for 6–8 h [15,16]. Oxidation was verified by determining the lipid peroxide content [15,16], and the oxidation degree was evaluated by measuring the relative electromobility rate on agarose gels as previously described [15,16].

### Cell culture and preparation of isolated islets

The insulin-secreting cell line (MIN6) was maintained as previously described [15,16]. Isolated human islets were obtained from the biotherapies for diabetes unit from the «Centre Hospitalier Régional et Universitaire de Lille». Human pancreatic tissue was harvested from brain-dead adult human donors in the context of the traceability requirements for the clinical islet transplantation program of the laboratory INSERM UMR 1190, Lille, France (clinicaltrials.gov, NCT01123187, NCT00446264, NCT01148680). The experimental design was approved in agreement with French regulations, our Institutional Ethical Committee of the University of Lille and the Centre Hospitalier Régional Universitaire de Lille. Informed consent from the next of kin, is obtained on the behalf of the deceased by the National French Procurement Agency «Agence de la BioMedecine». Consent was verbal. In France, it is presumed that you agree to organ donation unless you have expressed your refusal in the National Registry for Refusal of organ donation (NRR). The Agence de la BioMedecine Coordinators ask for verbal consent and then consult the NRR (ie written refusal). Therefore the written refusal is based on the NRR. None of the transplant donors were from a vulnerable population and all donors or next of kin provided both informed verbal consent and written consent after consulting the NRR that was freely given. As a direct result of informed consent for scientific research, we check a box for each donor on our human islet data base autorization for Research obtained YES or NO. Human islets that did not receive prior informed consent for Science are never used for research. Isolation and preparation of islets in Lille were conducted as described previously [24]. Experiments were carried out on fresh human islets preparation with >80% viability and >80% purity. After isolation, human islets were cultured in CMRL-1066 medium culture containing 5 mmol/l glucose, 100 U/ml penicillin, 100 μg/ml streptomycin and 10% fetal bovine serum (Mediatech Herndon, VA) in 5% CO_2_ humidified atmosphere at 37°C. For measuring gene expression and insulin secretion in response to lipoproteins, 200 and 40 islets equivalent number (IE) were plated in 24-well plate, respectively.

### Apoptosis assay

Apoptosis was determined by scoring cells displaying pyknotic nuclei (visualized with Hoechst 33342) [15,16]. The counting was performed blind by three different experimenters.

### Western blotting, total RNA preparation and real-time PCR

For Western blotting, total protein extracts were separated by SDS-PAGE and blotted on nitrocellulose membranes as described [15,16]. The proteins were detected using specific antibodies and were visualized with IRDye 800 (Rockland) as secondary antibodies and quantified with the Odyssey Infrared Imaging System (Li-COR). Total RNA from insulin-secreting cell lines and pancreatic islets was extracted using phenol/chloroform extraction according to Chomczynski and Sacchi’s protocol. Reverse transcription reactions were performed as previously described [15,16]. Real-time PCR assays were carried out on a BioRad MyiQ Single-Color Real-Time PCR Detection System using the BioRad iQ SYBR Green Supermix (Bio-Rad Laboratories, CA, USA) exactly as previously described [12, 13]. Primer sequences of mouse preproinsulin 2 (*Ins2*), human preproinsulin, *Crem/CREM* and *Rplp0/RPLP0* are those published elsewhere [12, 13]. The human and mouse sequences of PCR primers were as follows: For *Chop* mouse primers: forward: 5’-TTCACTACTCTTGACCCTGCGT-3’ and reverse 5’- CACTGACCACTCTGTTTCCGTTTC-3’; Mouse *Atf4*: forward: 5’- ATCCAGCAAAGCCCCACAAC-3’ and reverse 5’-CAAGCCATCATCCATAGCCG-3’; mouse *p58IPK*: forward 5’-AAGCCCGTGGAAGCCATTAG-3’ and reverse 5’-GGTCATTTTCATTGTGCTCCTGAG-3’; The primer sequences for human *CHOP* were forward: 5’-GTGAATCTGCACCAAGCATGA-3’ and reverse 5’-AAGGTGGGTAGTGTGGCCC-3’; human *ATF4*: forward: 5’- TGGCTGGCTGTGGATGG-3’ and reverse 5’-TCCCGGAGAAGGCATCCT-3’.

### Statistical analyses

The experiments including more than two groups were analyzed by ANOVA or with the non-parametric equivalent Kruskal-Wallis test. Appropriate corrections such as Tukey's and Bonferroni's *post hoc* tests were used for multiple comparisons. Statistical analyses were performed using GraphPad PRISM, 5.0 (GraphPad Software, La Jolla, California, USA)

## Results

### Human oxidized LDL particles activate the Ire1α and ATF6 pathways in MIN6 cells and isolated human islets

**Fig 1:**
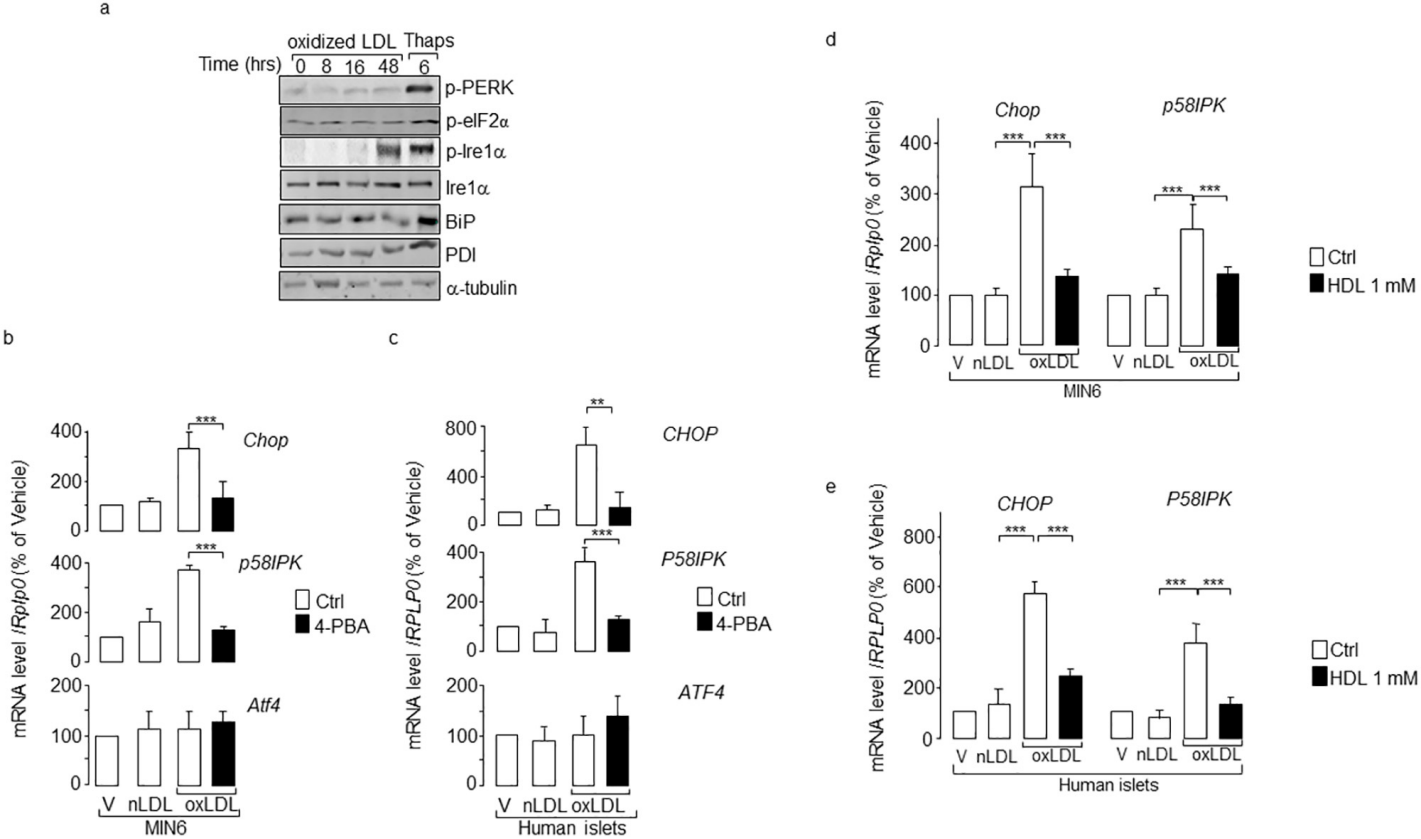
Activation of ER stress by human oxidized LDL. **(a)** Western blotting analysis comparing changes in PERK, eIF2 and Ire1α and their phosphorylated forms (p). Total proteins were prepared from MIN6 cells cultured with 2 mmol/l cholesterol oxidized LDL (oxLDL) for the indicated times and 1 μmol/l thapsigargin (Thaps) for 6 h. The α-tubulin protein served as loading control. The figure is a representative experiment out of three. Measurement of *CHOP*/*Chop*, *P58IPK*/*p58IPK* and *ATF4*/*Atf4* mRNA levels in (**b)** and **(d)** MIN6 and (**c)** and **(e)** isolated human islets cells cultured with oxidized LDL. The mRNA level was quantified by quantitative real-time PCR in MIN6 or isolated human islets cells cultured for 48 h with vehicle (V), native (nLDL) or oxidized LDL (oxLDL). The PBA chemical chaperone 2.5 mmol/l were added in the cells cultured with oxidized LDL (oxLDL, *filled bar*). For **d**) and **e**), cells were cultured with oxidized LDL plus 1 mmol/l cholesterol HDL (*filled bar*). The mRNA level was normalized against the housekeeping acidic ribosomal phosphoprotein P0 gene (*RPLP0*/*Rplp0*) and the expression levels from cells cultured with vehicle were set to 100%. Data are the mean of ± SEM of 3 independent experiments performed in triplicate (***, P<0.001; **, P<0.01).

To investigate whether the induction of ER stress contributes to the adverse effects of human oxidized LDL, MIN6 cells were incubated with human mildly oxidized LDL at a 2 mmol/l cholesterol concentration and at different incubation times. As a positive control for ER stress, MIN6 cells were cultured for 6 h with Thapsigargin (thaps), a sarcoendoplasmic-reticulum Ca^2+^-ATPase pump inhibitor [25]. As expected, we found that this chemical compound activated PERK and increased the phosphorylation of its direct substrate, eukaryotic translation initiation factor 2 subunit alpha (eIF2α) (Fig 1a). The ER stress sensor Ire1α was phosphorylated by thaps in MIN6 cells (Fig 1a). Phosphorylation of Ire1α also occurred in response to oxidized LDL, whereas the induction of the PERK pathway was not detectable in this context (Fig 1a). In support of Ire1α activation, splicing of *Xbp1* increased in MIN6 cells cultured with oxidized LDL for 48 h (S1a Fig). When the unfolded protein response (UPR) is activated, the expression of heat shock protein family A (Hsp70) member 5 (HSPA5, also known as BiP) and Protein Disulfide Isomerase (PDI) is known to be increased [19,26]. In this regard, we found that UPR induced by thaps was associated with an increase in BiP and PDI expression in pancreatic beta-cells (Fig 1a), but this was not seen when the cells were exposed to oxLDL (Fig 1a and S1b Fig).

If the ER stress is severe and chronic, the DNA damage inducible transcription factor 3 (DDIT3; also known as CHOP) expression is known to be increased, leading to apoptosis [27,28]. Change in the expression of CHOP was found to be associated with diabetes [20, 21] and the genetic ablation of *Chop* in mice can delay the development of diabetes [28]. Here, consistent with the activation of Ire1α, *Chop/CHOP* expression was increased in isolated human islets and MIN6 cells exposed to oxidized LDL (Fig 1b and 1c). As expected, induction of *Chop* mRNA by oxidized LDL was followed by the increase of the protein (S1c Fig). In contrast, the expression of *Chop* was not stimulated by 2 mmol/l native LDL-cholesterol (Fig 1b and 1c), which is in support of the harmless effect of the native LDL at this concentration that we have previously reported [15,16]. Activation of Atf6 is known to lead to an increase in DnaJ (Hsp40) homolog, subfamily C, member 3 (*Dnajc3*, also called *p58IPK*) mRNA level in response to ER stress inducers [29]. Here, the oxidized LDL elicited a three-four -fold elevation in *p58IPK/P58IPK* mRNA in MIN6 and isolated human islets cells (Fig 1b and 1c), respectively, thus mirroring induction of the ATF6 pathway. However, the human modified LDL did not induce the expression of *Atf4/ATF4*, suggesting that the PERK pathway is not activated by oxidized LDL (Fig 1b and 1c). The chemical chaperone 4-phenyl butyrate (PBA) improves the folding capacity and trafficking of mutant proteins out of the ER and thereby reduces the load of unfolded proteins in the ER [30]. As anticipated, the induction of *Chop/CHOP* and *p58IPK/P58IPK* mRNA by oxidized LDL was efficiently blocked in MIN6 and isolated human islet cells that were co-cultured with PBA (Fig 1b and 1c). HDL has been shown to protect against ER stress [26,31]. Co-culture of MIN6 and human islet cells with HDL and oxidized LDL abolished the elevation of *Chop/CHOP* and *p58IPK/P58IPK* elicited by the oxidized LDL (Fig 1d and 1e). This result suggests that the induction of ER stress plays a key role in the deleterious effects caused by oxidized LDL.

### PBA partially prevents decreased insulin expression and apoptosis caused by oxidized LDL

**Fig 2:**
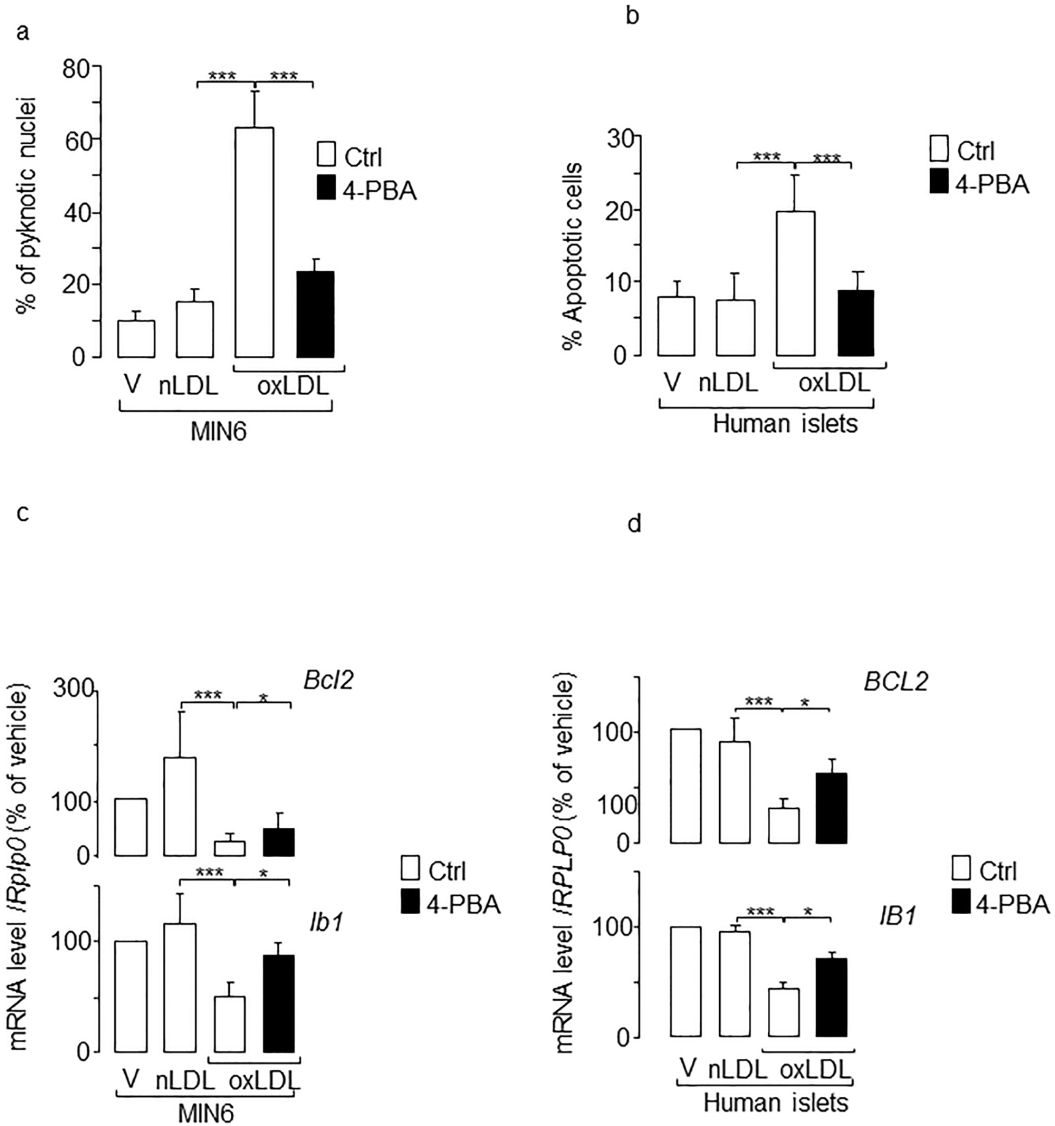
Effects of BPA on apoptosis evoked by human oxidized LDL. Measurement of apoptotic cells in **(a)** MIN6 and **(b)** isolated human islets. MIN6 cells were cultured with vehicle (V), native LDL (nLDL) or oxidized LDL (oxLDL) 2 mmol/l cholesterol with or without PBA 2.5 mmol/l (filled bar) for 72 h. The fraction of cells undergoing apoptosis was determined by scoring the percentage of cells displaying pyknotic nuclei. Data are the mean of ± SEM of 3 independent experiments (***, P<0.001; *, P<0.05). Quantification of the anti-apoptotic *Bcl2/BCL2* and *Ib1*/*IB1* mRNA levels in **(c)** MIN6 and **(d)** isolated human islets. The mRNA level of the two genes was quantified by quantitative real-time PCR and was normalized against the *Rplp0/RPLP0*. The expression levels from cells cultured with vehicle were set to 100%. Data are the mean of ± SEM of 3 independent experiments performed in triplicate (***, P<0.001; *, P<0.05).

**Fig 3:**
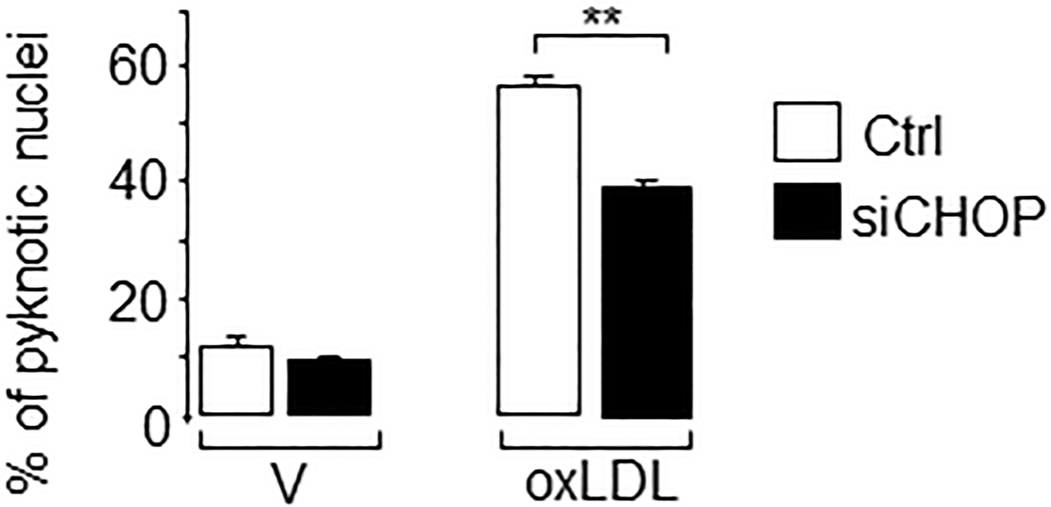
Effects of Chop silencing in apoptosis caused by human oxidized LDL. MIN6 cells were transfected with a control RNA si-GFP duplex (Ctrl, *open bar*) or with siCHOP (*filled bar*). Thereafter, the cells were cultured for 72 h with vehicle (V) or oxidized LDL (oxLDL) 2 mmol/l cholesterol. The fraction of cells undergoing apoptosis was determined by scoring the percentage of cells displaying pyknotic nuclei. Data are the mean of ± SEM of 3 independent experiments performed in triplicate (**, P<0.01).

We next investigated whether the activation of ER stress links oxidized LDL to beta cell dysfunction and death. We found a significant reduction in the number of pyknotic nuclei in MIN6 and isolated human islet cells that were co-cultured with the chemical chaperone 4-phenyl butyrate (PBA) (Fig 2a and 2b). Oxidized LDL-mediated apoptosis results in part from decreased anti-apoptotic *Mapk8ip1 (*also called *Ib1)* and *Bcl2* genes expression [12, 13]. Here, the protective effect triggered by PBA was accompanied by a partial restoration in the expression of *Ib1* and *Bcl2* (Fig 2c and 2d), confirming the anti-apoptotic action of PBA against oxidized LDL. Apoptosis caused by oxidized LDL is known to involve *Chop/CHOP* [32]. Indeed, we found that the silencing of Chop by siRNAs (S2 Fig) reduced pancreatic beta-cell death induced by oxidized LDL (Fig 3).

**Fig 4:**
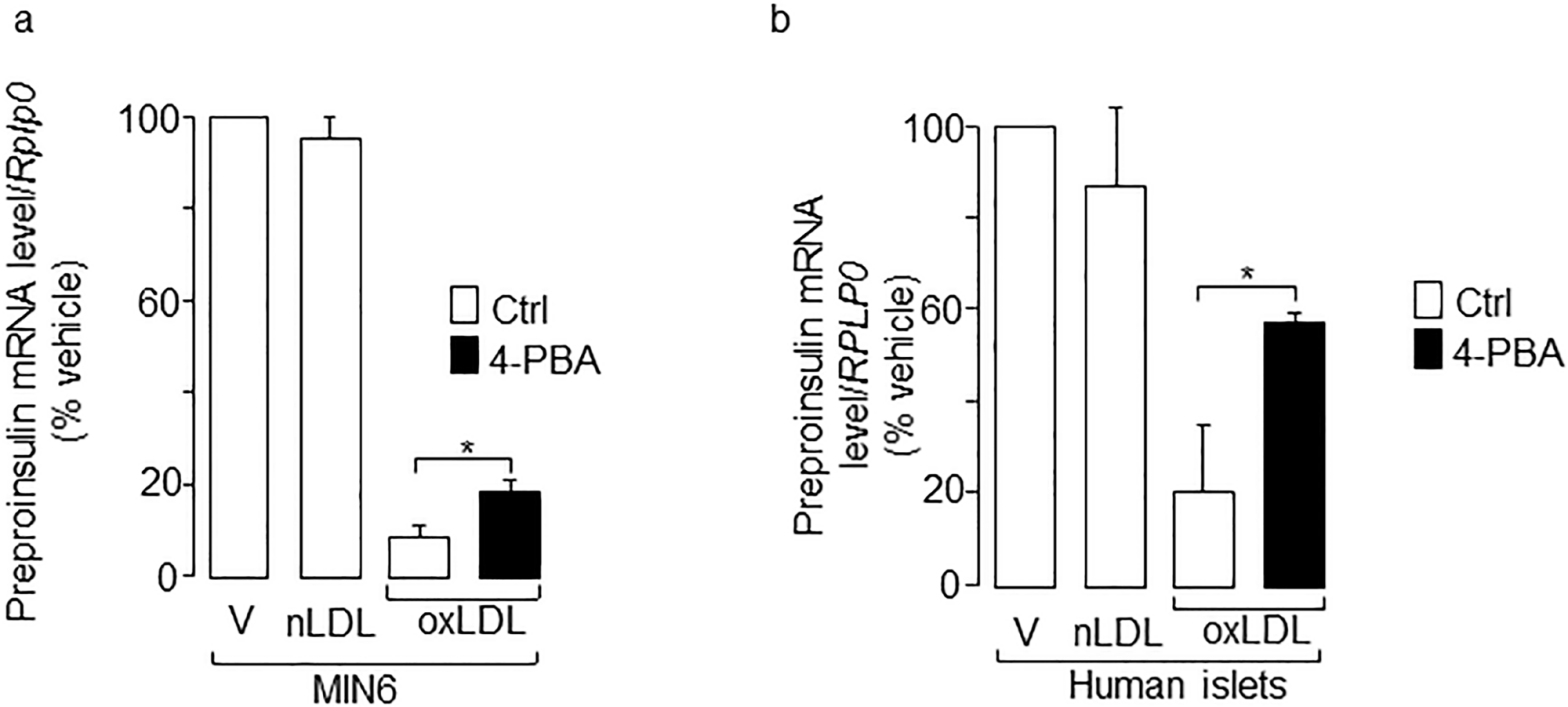
Effects of 4-BPA chemical chaperone on the loss of insulin expression caused by human oxidized LDL. The preproinsulin mRNA was quantified in MIN6 cells (**a)** and (**b**) human islets. Cells were exposed to vehicle (V), human native LDL (nLDL) or oxidized LDL (oxLDL) 2 mmol/l cholesterol, in the presence or absence of PBA 2.5 mmol/l (filled bars) for 48 h. The mRNA levels were normalized against the *Rplp0/RPLP0* and the expression levels from cells cultured with vehicle were set to 100%. Data are the mean of ± SEM of 3 independent experiments performed in triplicate (*, P<0.05).

**Fig 5:**
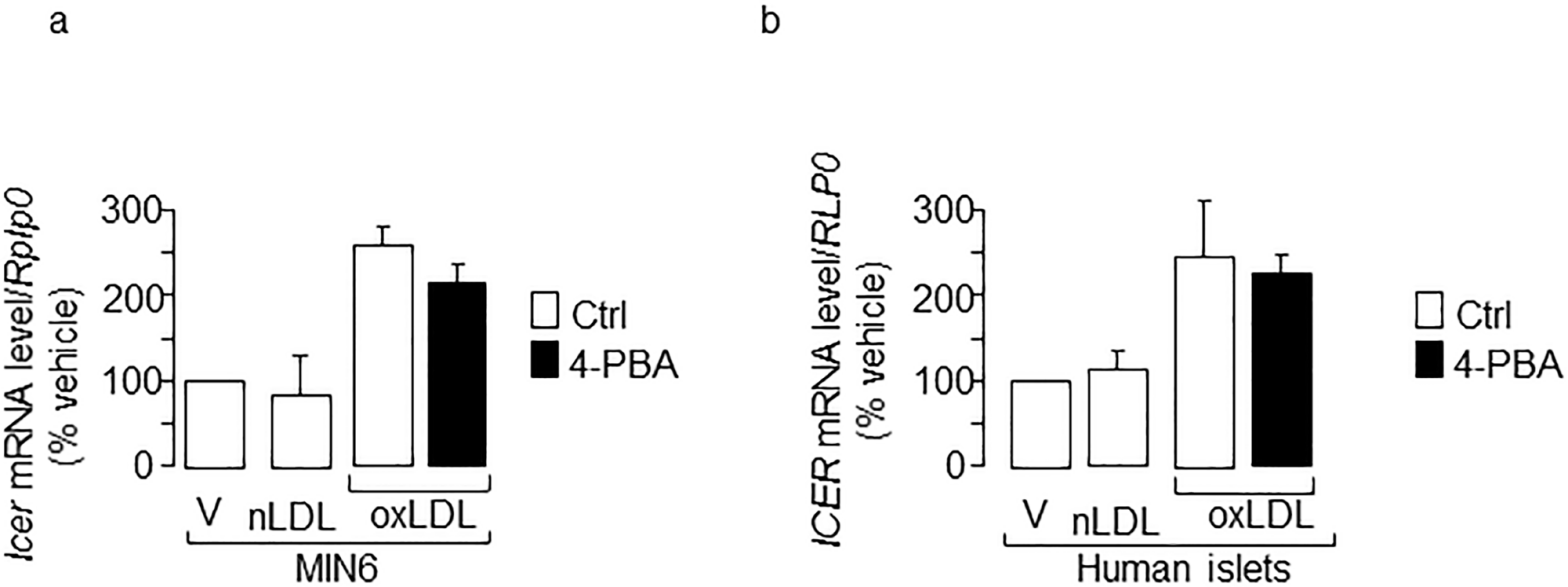
Effects of 4-BPA chemical chaperone on the induction of Icer/ICER mRNA evoked by human oxidized LDL. Quantification of *Icer*/*ICER* mRNA levels in response to vehicle (V), human native LDL (nLDL) or oxidized LDL (oxLDL) 2 mmol/l cholesterol in (**a)** MIN6 and (**b**) human islets. The expression levels from total islets or cells cultured with vehicle were set to 100%. Data are the mean of ± SEM of 3 independent experiments performed in triplicate.

Beside apoptosis, oxidized LDL is known to impair insulin gene expression. Indeed, the loss of insulin expression appears earlier than both insulin secretion deficiency and cell death [15,16]. To determine whether the induction of ER stress by oxidized LDL contributes to the impaired insulin expression, MIN6 and isolated human islet cells were cultured with lipoproteins with or without PBA. Inhibition of the ER stress markers by PBA were accompanied by a partial restoration of insulin mRNA levels, indicating a role for ER stress in the deleterious effect of oxidized LDL (Fig 4a and 4b). Oxidized LDL have been shown to hamper glucose-induced insulin secretion [16]. However, in our study, insulin secretion was not rescued by PBA co-treatment (data not shown), suggesting that induction of ER stress by the modified lipoproteins is not involved in the impaired insulin secretion. In addition to ER stress, the induction of CREM (also called as inducible cAMP early repressor; ICER) also accounts for the loss of insulin production, impaired glucose-induced insulin secretion and decline in beta-cell survival provoked by oxidized LDL [16]. However, in our study PBA efficiently alleviated the increase of ER stress markers and apoptosis, thus the chemical chaperone was unable to antagonize the oxidized LDL-induced augmentation of *Icer*/*ICER* in MIN6 and isolated human islets (Fig 5a and 5b). These results indicate that induction of Icer by oxidized LDL relies on mechanisms that do not involve ER stress.

### ER stress links oxidative stress to beta-cell dysfunction caused by human oxidized LDL

**Fig 6:**
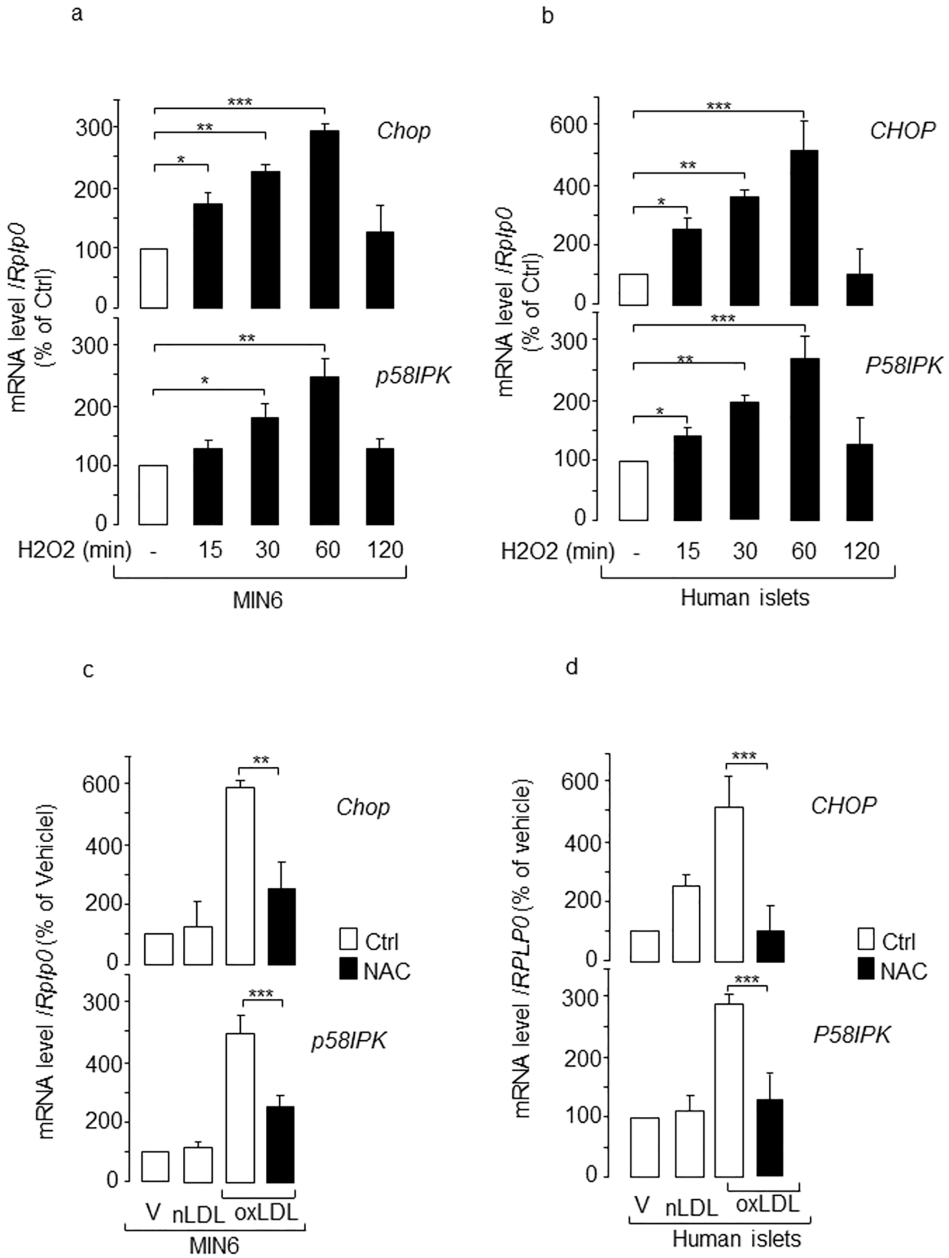
Role of oxidative stress on the ER stress markers expression induced by human oxidized LDL. Expression of *CHOP*/*Chop* and *P58IPK*/*p58IPK* in **(a)** MIN6 and **(b)** human islets cells exposed to hydrogen peroxide (H_2_O_2_) 150 μM at indicated times. The expression of the two genes was quantified in **(c)** MIN6 cells or **(d)** human islets that were co-incubated with vehicle (V), human native LDL (nLDL) or oxidized LDL (oxLDL) 2 mmol/l cholesterol supplemented by either DMSO (control, open bar) or N-acetylcystein (NAC, filled bar) 1 mmol/l for MIN6 and 10 mmol/l for human islets cells. The results were normalized against *Rplp0*/*RPLP0* and the expression levels from cells cultured with vehicle were set to 100%. Data are the mean ± SEM of 3 independent experiments performed in triplicate (***, P<0.001; **, P<0.01).

Beta cell exposed to oxidized LDL produces hydrogen peroxide [16]. Treatment with the antioxidant N-acetylcystein (NAC) antagonizes the loss of insulin production, insulin secretion and beta-cell death provoked by oxidized LDL [16]. Therefore, we sought to determine whether ER stress couples oxidative stress to beta-cell dysfunction and death caused by oxidized LDL. This link is further supported by our observation that exposure of MIN6 cells and isolated human islets to hydrogen peroxide rapidly increased *Chop/CHOP* and *p58IPK/P58IPK* mRNA levels (Fig 6a and 6b). In fact, the co-culture of MIN6 cells and isolated human islets with NAC prevented the increase in *Chop/CHOP* and *p58IPK/P58IPK* mRNA by oxidized LDL (Fig 6c and 6d).

## Discussion

In this study, we evaluated the contribution of human oxidized lipoproteins, at the concentrations observed in sera of atherogenic dyslipidemic patients on ER stress signaling. We found that mildly oxidized LDL induced IRE1α signaling. Typically the IRE1α branch elicits mitogen-activated protein kinase 8 MAPK8 (also called JNK1) activity and *Chop/CHOP* expression [21,33,34]. Further, induction of IRE1α is consistent with the previously described activation of MAPK8 in beta-cells exposed to oxidized LDL [13]. The rise of CHOP content favors beta-cell apoptosis [28]. In our study, Chop silencing indeed attenuated beta-cell death caused by the modified LDL. Thus, the activation of IRE1α signalling may contribute to apoptosis evoked by oxidized LDL. The level of Chop can also be stimulated by the PERK pathway. Once activated PERK phosphorylates eIF2α, thus activating ATF4 [34]. In turn, ATF4 stimulates the expression of Chop [34]. The contribution of PERK pathway to the induction of Chop is unlikely as the PERK pathway was not activated by oxidized LDL. The activation of ATF6 triggers the expression of P58IPK. The latter is a cytosolic inhibitor of PERK activity, which is thought to be critical for regulating the latter phase of the ER stress response [35]. The expression of *P58IPK* increased in response to oxidized LDL. Our result suggests that the induction of *P58IPK* by oxidized LDL inhibits the induction of PERK.

**Fig 7:**
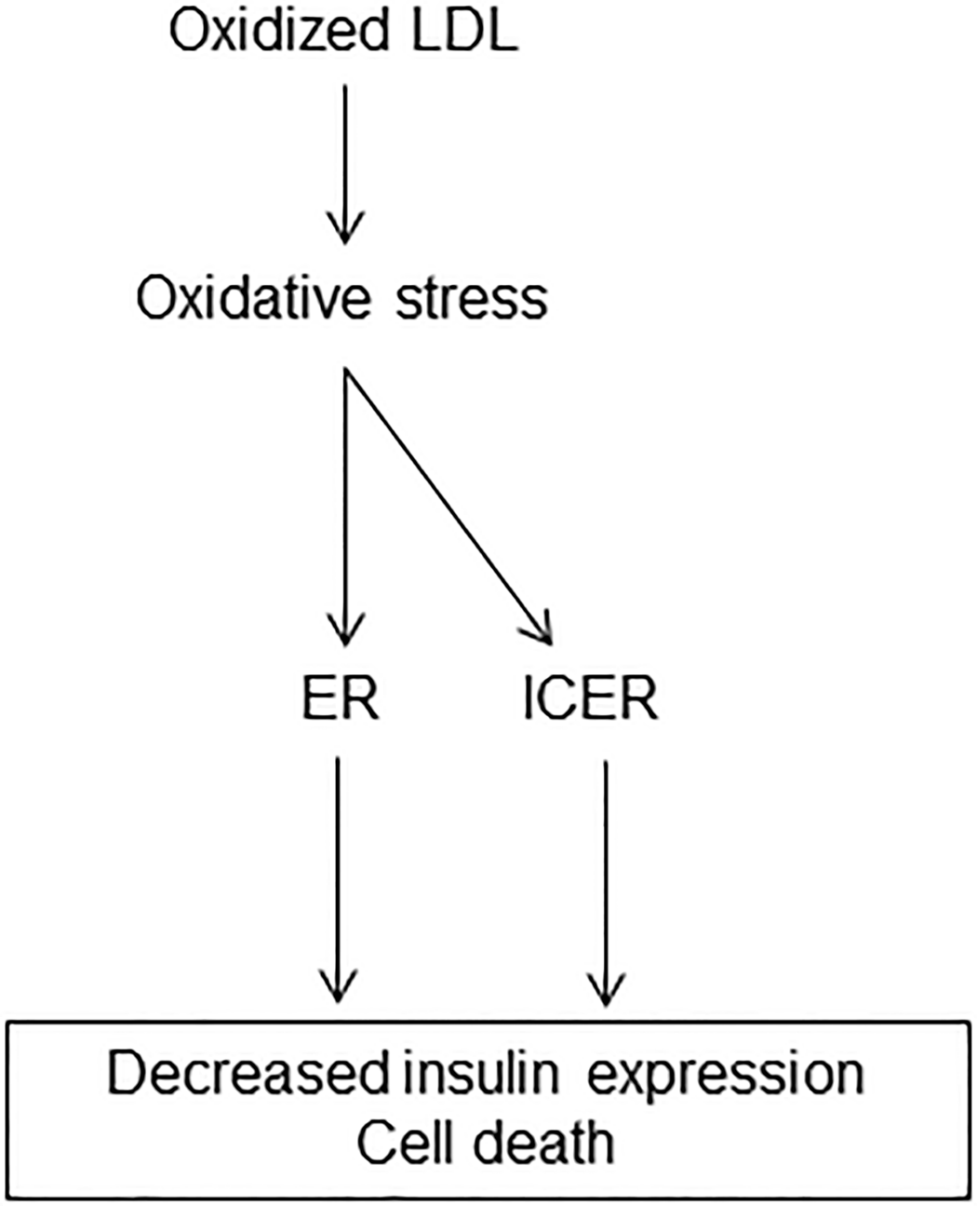
Schematic representation of mechanism coupling oxidized LDL to beta cell dysfunction and death.

Beside the ER stress activation, we have previously shown an increase in the expression of ICER passive transcriptional repressor in isolated islets and insulin producing cells cultured with oxidized LDL [16]. Although increased level of ICER correlated with this of Chop, our data rule out a link between ER stress and ICER. While the elevation of the ER stress marker was attenuated by PBA, induction of ICER by oxidized LDL remained elevated in cells cultured with the chaperone. A major target of ICER is IB1 [36]. ICER competes with cAMP-dependent transcriptional activators including cAMP response element (CRE) binding protein 1 (CREB1) for binding the CRE within target genes [37]. As the result of ICER binding, CREB1 target gene expression is silenced [16]. The loss of the *IB1* transcript by Icer is one of the mechanisms that couples oxidized LDL to beta-cell dysfunction and death [15,16]. Treatment of cells with the PBA partially prevented the loss of *Ib1* mRNA. This result suggests that the loss of *IB1* expression, and thereby the reduced insulin expression and apoptosis induced by oxidized LDL, rely on two independent mechanisms, one dependent of Icer and another one that involves the ER stress (Fig 7).

ER stress is a hallmark of beta-cell damage in T2D [30]. In this study, we unveiled that NAC efficiently antagonized the elevation of ER stress markers in response to oxidized LDL. We have previously shown activation of oxidative stress in beta-cells exposed to oxidized LDL, leading to peroxide formation [16]. Production of reactive oxygen species is known to cause damages to DNA, proteins, sugars, and lipids [38]. Modification of proteins by reactive species may lead to misfolded proteins and thereby, induce ER stress in beta cell exposed to oxidized LDL. Our findings strengthen the hypothesis that the supplementation with antioxidants may help in combating beta-cell failure in diabetes and its secondary cardiovascular complications. In a meta-analysis study, vitamin E and carotenoids antioxidant intake was associated with a reduced T2D risk [39]. Our data highlight a link between oxidative stress and ER stress, which could be efficiently antagonized by antioxidant treatments.

## Supporting Information

S1 Fig mRNA level of ER stress markers in response to oxidized LDL. Quantification of **(a)** *Xbp1* mRNA splicing and **(b)** *Bip* in MIN6 cells exposed to oxidized LDL. The mRNA level was quantified by quantitative real-time PCR in MIN6 cells cultured for 48 h with vehicle (V), 2 mmol/l cholesterol native (nLDL) or oxidized LDL (oxLDL). *Xbp1* cDNA was amplified by PCR and digested with *Pst*I enzyme. Spliced *Xbp1* cDNA corresponds to the activated form. This form lacks the restriction site and consequently remains intact. Spliced and unprocessed *Xbp1* was quantified by densitometry. The value obtained for processed *Xbp1* was expressed as a ratio of the total *Xbp1* mRNA levels for each sample. The expression of Bip was normalized against *Rplp0* and the expression levels from cells cultured with vehicle were set to 100%. The ratio from cells cultured with vehicle was set to 100%. Data are the mean ± SEM of at least 3 independent experiments measured in triplicate (*, P<0.05). (**c**) Western blotting analysis of Chop in MIN6 cells cultured with oxidized LDL. Total proteins were prepared from MIN6 cells cultured with 2 mmol/l cholesterol oxidized LDL (oxLDL) for the indicated times and 1 μmol/l thapsigargin (Thaps) for 6 h. The α-tubulin protein served as loading control. The figure is a representative experiment out of three.
(PPTX)

S2 Fig Efficiency of Chop silencing by small interfering RNAs. MIN6 cells were either transfected with duplexes of control small interfering directed specifically against GFP (Ctrl, open bar) or siRNA directed against Chop (siCHOP, filled bar). Thereafter, the cells were cultured for 72 h with vehicle (V) or 2 mmol/l cholesterol oxidized LDL (oxLDL). The mRNA level was normalized against the *Rplp0* and the expression levels from cells cultured with vehicle were set to 100%. Data are the mean of ± SEM of 3 independent experiments (***, P<0.001).
(PPTX)

## Abbreviations

BiP: Immunoglobulin heavy chain-Binding Protein
CHOP: CAAT/enhancer-binding protein homologous protein
eIF2a: eukaryotic initiation factor 2α
ER: Endoplasmic Reticulum
IB1: Islet Brain 1
ICER: Inducible cAMP early repressor
Ire1α: Inositol requiring 1α
NAC: N-AcetylCystein
PBA: PhenylButyric Acid
PERK: Eukaryotic translation initiation factor 2-alpha kinase 3
UPR: Unfolded Protein Response
XBP1: X-Box binding protein 1
