# Supplementary material for: Endoplasmic Reticulum Stress Links Oxidative Stress to Impaired Pancreatic Beta-Cell Function Caused by Human Oxidized LDL
Source: PLoS One. 2016 Sep 16;11(9):e0163046. doi: 10.1371/journal.pone.0163046 (PMC5026355; doi:10.1371/journal.pone.0163046)
Supplement: S1 Fig — Quantification of (a) Xbp1 mRNA splicing and (b) Bip in MIN6 cells exposed to oxidized LDL. The mRNA level was quantified by quantitative real-time PCR in MIN6 cells cultured for 48 h with vehicle (V), 2 mmol/l cholesterol native (nLDL) or oxidized LDL (oxLDL). Xbp1 cDNA was amplified by PCR and digested with PstI enzyme. Spliced Xbp1 cDNA corresponds to the activated form. This form lacks the restriction site and consequently remains intact. Spliced and unprocessed Xbp1 was quantified by densitometry. The value obtained for processed Xbp1 was expressed as a ratio of the total Xbp1 mRNA levels for each sample. The expression of Bip was normalized against Rplp0 and the expression levels from cells cultured with vehicle were set to 100%. The ratio from cells cultured with vehicle was set to 100%. Data are the mean ± SEM of at least 3 independent experiments measured in triplicate (*, P<0.05). (c) Western blotting analysis of Chop in MIN6 cells cultured with oxidized LDL. Total proteins were prepared from MIN6 cells cultured with 2 mmol/l cholesterol oxidized LDL (oxLDL) for the indicated times and 1 μmol/l thapsigargin (Thaps) for 6 h. The α-tubulin protein served as loading control. The figure is a representative experiment out of three. (PPTX) [file pone.0163046.s001.pptx]

## Slide 1
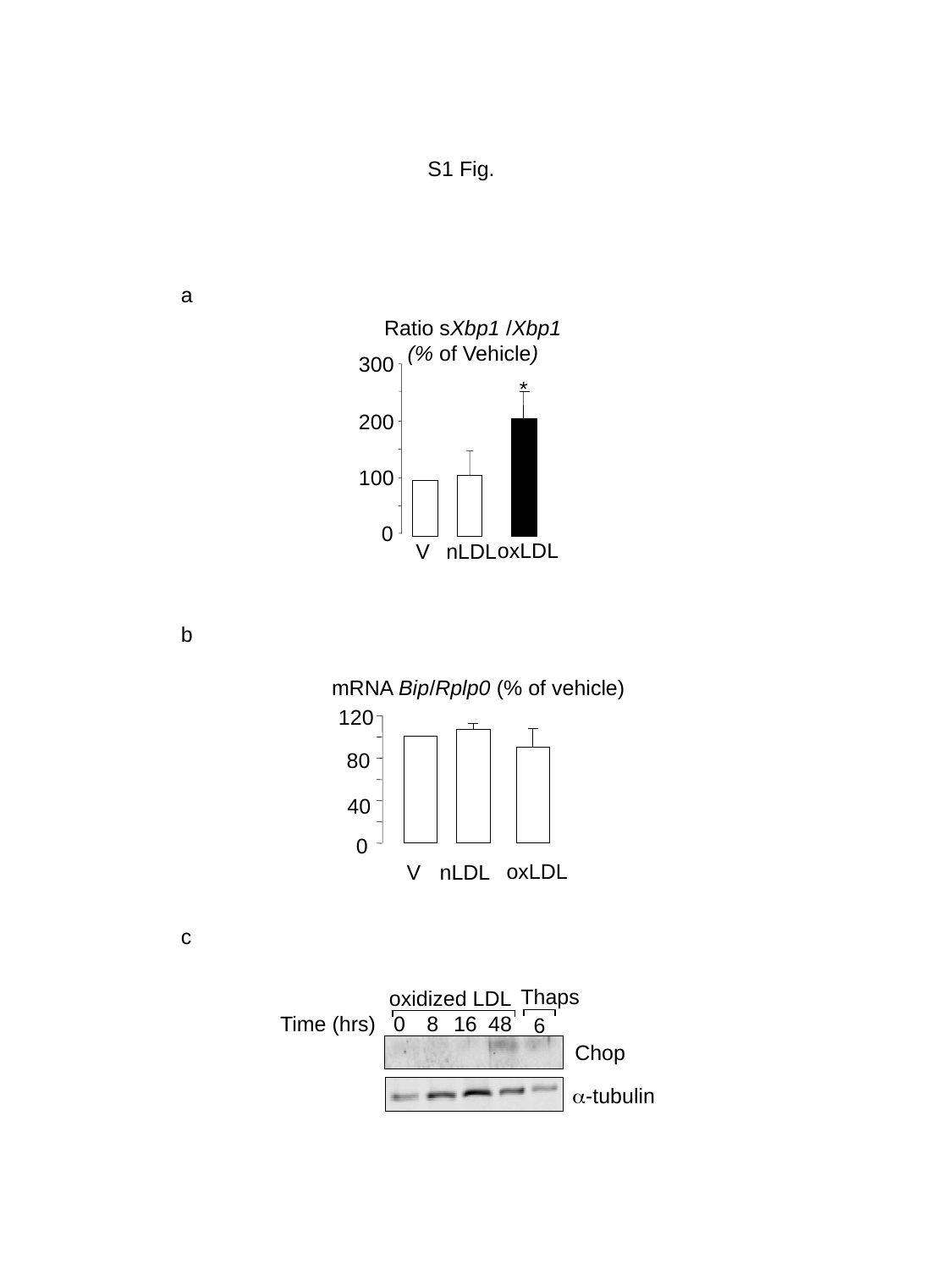

S1 Fig.
a
Ratio sXbp1 /Xbp1
(% of Vehicle)
300
*
200
100
0
oxLDL
V
nLDL
b
mRNA Bip/Rplp0 (% of vehicle)
120
80
40
0
oxLDL
nLDL
V
c
Thaps
oxidized LDL
Time (hrs)
0
8
16
48
6
Chop
a-tubulin
