# Supplementary figures and images for: Endoplasmic Reticulum Stress Links Oxidative Stress to Impaired Pancreatic Beta-Cell Function Caused by Human Oxidized LDL
Source: PLoS One. 2016 Sep 16;11(9):e0163046. doi: 10.1371/journal.pone.0163046 (PMC5026355; doi:10.1371/journal.pone.0163046)

## Slide 1
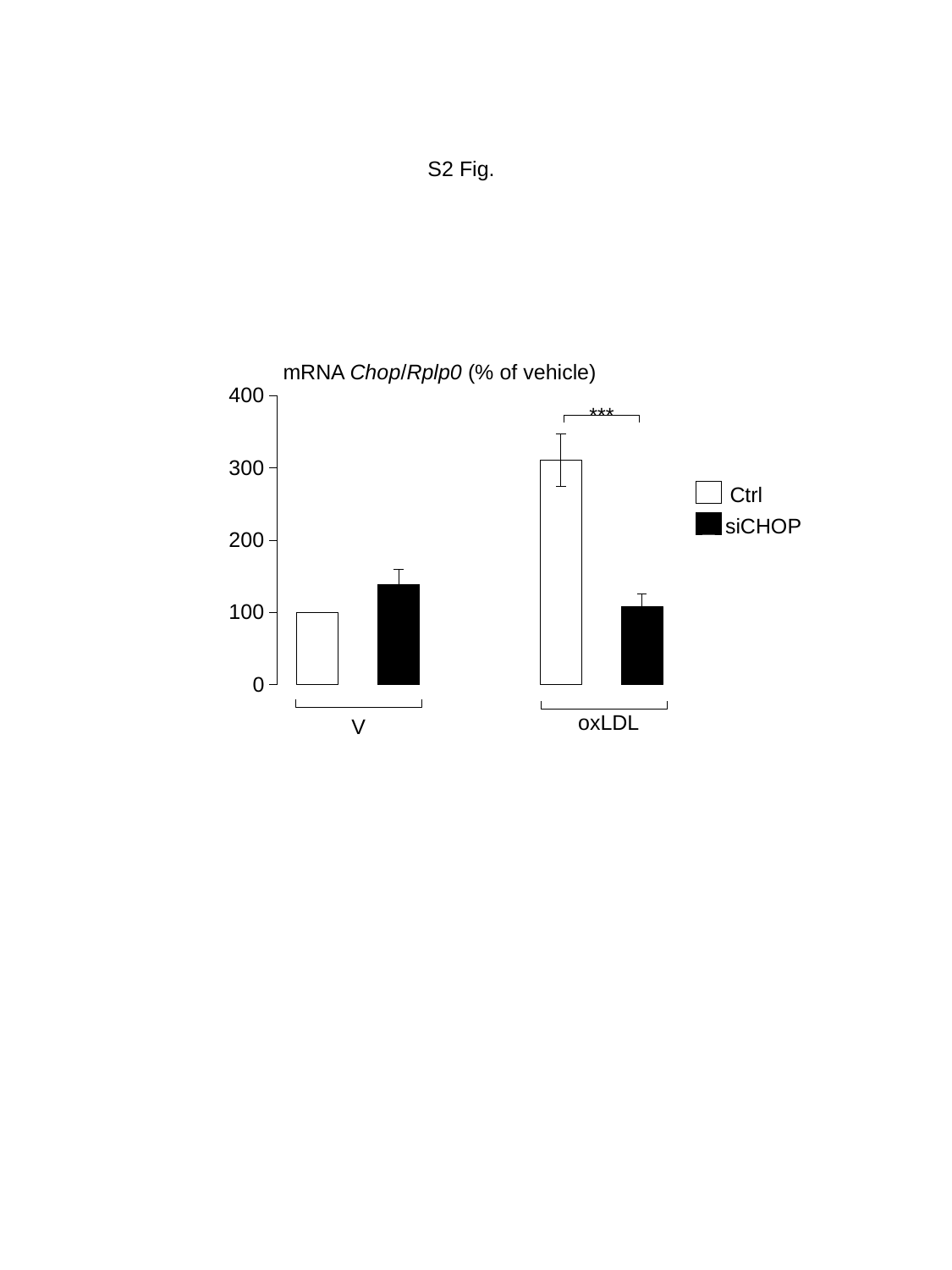

S2 Fig.
mRNA Chop/Rplp0 (% of vehicle)
### Chart
| Category | |
|---|---|
***
Ctrl
siCHOP
oxLDL
V

Supplement: S2 Fig — MIN6 cells were either transfected with duplexes of control small interfering directed specifically against GFP (Ctrl, open bar) or siRNA directed against Chop (siCHOP, filled bar). Thereafter, the cells were cultured for 72 h with vehicle (V) or 2 mmol/l cholesterol oxidized LDL (oxLDL). The mRNA level was normalized against the Rplp0 and the expression levels from cells cultured with vehicle were set to 100%. Data are the mean of ± SEM of 3 independent experiments (***, P<0.001). (PPTX) [file pone.0163046.s002.pptx]
